# Aqueous exposure to a pyrethroid pesticide results in behavioural effects in early life stage sturgeon

**DOI:** 10.1093/conphys/coaf055

**Published:** 2025-07-30

**Authors:** Anna E Steel, Sarah E Baird, Dennis E Cocherell, Thomas M Young, Richard E Connon, Nann A Fangue

**Affiliations:** Department of Wildlife, Fish, and Conservation Biology, University of California, Davis, One Shields Ave, Davis, CA, 95616 USA; Department of Wildlife, Fish, and Conservation Biology, University of California, Davis, One Shields Ave, Davis, CA, 95616 USA; Department of Wildlife, Fish, and Conservation Biology, University of California, Davis, One Shields Ave, Davis, CA, 95616 USA; Department of Civil and Environmental Engineering, University of California, Davis, One Shields Ave, Davis, CA, 95616 USA; Department of Anatomy, Physiology and Cell Biology, School of Veterinary Medicine, University of California, Davis, One Shields Ave, Davis, CA, 95616 USA; Department of Wildlife, Fish, and Conservation Biology, University of California, Davis, One Shields Ave, Davis, CA, 95616 USA

**Keywords:** Behavioural response, bifenthrin, pyrethroid toxicity, sturgeon larvae, sublethal effects

## Abstract

The presence of chemical contaminants in freshwater systems poses a threat to many aquatic organisms, and understanding the extent and nature of this threat can facilitate conservation management actions. Sturgeon are considered threatened worldwide and they differ in many important ways from other fishes. Two sturgeon species, green sturgeon (*Acipenser medirostris*) and white sturgeon (*A. transmontanus*), are found in California and utilize anthropogenically impacted freshwater habitats of the Central Valley. This study evaluated the behavioural effects in endogenously feeding larvae (3–7 days post hatch) of both sturgeon species following an acute exposure (96 hours) to the pyrethroid pesticide bifenthrin at aqueous concentrations ranging from 10 to 2000 ng/l, with selected levels based on previous environmental monitoring. Sturgeon had high survival at all concentrations tested (~95%), yet at higher concentrations (>1000 ng/l) they displayed altered behavioural patterns, including reduced activity, increased meander of the movement path and reduced thigmotaxis. While these higher concentrations of bifenthrin have been observed within water samples from the sturgeon habitats of California, they appear uncommon. The present study suggests that sturgeon larvae are not highly sensitive to acute aqueous exposure under environmentally relevant concentrations of bifenthrin (1–10 ng/l), yet these aqueous concentrations do have behavioural effects that may be of concern for the conservation of these declining species. Additionally, impacts to these species may also occur through exposure to sediment-bound bifenthrin or dietary bioaccumulation, and more work needs to be done to understand the implications of these exposure routes.

## Introduction

Anthropogenic activity over the last 150 years has severely disrupted freshwater habitats, threatening fish and other species (Lotze *et al.*, 2006; Moyle *et al.*, 2010). One of the many transformations is the deterioration of water quality due to presence of chemical contaminants (Sommer *et al.*, 2007). Contaminants can range from heavy metals to synthetic pesticides, and exposure can result in both lethal and sublethal impacts eliciting acute or chronic physiological responses. Contaminants are a concern for conservation of declining species because they can reduce survival, lower fecundity, and impact population persistence over time (Willson *et al.*, 2012; Bourillon *et al.*, 2022). Laboratory studies have highlighted a multitude of mechanistic responses in fishes, resulting from exposure to contaminants (Connon *et al.*, 2009; Beggel *et al.*, 2010, 2011; Brander *et al.*, 2016; Fong *et al.*, 2016; Hutton *et al.*, 2023), yet effects on many species of conservation concern are understudied (Connon *et al.*, 2019; Cooke *et al.*, 2023).

Sturgeons are ancient ray-finned fish uniquely adapted to mainstem river systems and estuaries. Most of the extant sturgeon species are classified as vulnerable or endangered on the IUCN red list (IUCN, 2023). They are large-bodied, long-lived, late-maturing and slow-growing, and as such, have a life history that is susceptible to multiple anthropogenic threats (Beamesderfer and Farr, 1997; Billard and Lecointre, 2001; Israel *et al.*, 2008; Israel and Klimley, 2008; NMFS, 2009; Mussen *et al.*, 2014; Poletto *et al.*, 2014; Rodgers *et al.*, 2019). Previous research on contaminant effects on sturgeon have shown that this taxon is vulnerable to several contaminant types, including pesticides (Feist *et al.*, 2005; Calfee *et al.*, 2014; Dwyer *et al.*, 2020). The limited research that has been done on sturgeon species has shown that sturgeon sensitivity is generally comparable to those of many salmonids (Dwyer *et al.*, 2020). However, contaminant-specific toxicity can differ substantially across species, even within a taxon, and sensitivity can vary by life stage, with early life stages believed to be the most sensitive (Jahanbakhshi *et al.*, 2012; Calfee *et al.*, 2014).

Because sturgeon generally inhabit large river systems, their freshwater habitats worldwide are heavily impacted by anthropogenic influences, including chemical contaminants. In California, the San Francisco Estuary (SFE) includes high densities of agriculture and urban pesticide use (Weston and Lydy, 2010; Fong *et al.*, 2016) while also serving as critical habitat to green sturgeon (*Acipenser medirostris*) and white sturgeon (*A. transmontanus*). Both species utilize SFE habitats to support growth from larval through sub-adult stages (0–4 + yrs). While detailed studies of mercury and selenium uptake have been done with California sturgeon species (Lee *et al.*, 2011, 2012; Linares-Casenave *et al.*, 2015), there is little information on the effects of synthetic pesticides. The California Department of Pesticide Regulation’s (CDPR) Surface Water Protection Program has ranked pyrethroids as high priority for monitoring in the SFE because they have potential to cause surface water toxicity from urban and agricultural uses (Luo, 2014). Pyrethroid pesticides are highly toxic to fish, reportedly 1000 times more so than to mammals (Brander *et al.*, 2016), acting primarily as a neurotoxin by disrupting voltage-gated sodium channels and altering nerve signalling (Magnuson *et al.*, 2022). Pyrethroid metabolism and elimination are significantly slower in fish than in birds and mammals, potentially increasing bioaccumulation and driving the higher toxicity observed in fish (Yang *et al.*, 2020). Pyrethroids can have additional effects that can be detrimental to fish, including impacts to development, growth, behavioural alterations, osmoregulation and endocrine disruption (Connon *et al.*, 2009; Jeffries *et al.*, 2015; Brander *et al.*, 2016; Segarra *et al.*, 2021). Pyrethroid effects are also commonly assessed in recent aquatic ecotoxicity studies, providing a valuable metric for comparison of sensitivity across aquatic taxa (Ranatunga *et al.*, 2023).

In evaluations of sediments and surface water contaminants in freshwater tributaries of the SFE, the pyrethroid bifenthrin has been identified as posing high overall relative risk to aquatic life and is one of the most commonly detected pyrethroids in the SFE, frequently at aqueous concentrations at or above known toxicity levels (Amweg *et al.*, 2006; Sanders *et al.*, 2018; Weston *et al.*, 2019). Bifenthrin can enter surface waters through runoff from either urban or agricultural sources, as it is widely used in structural pest control, urban landscaping and pest control on many agricultural crops (Hanson *et al.*, 2022). Bifenthrin, a type I pyrethroid insecticide, targets voltage-gated sodium channels, resulting in constant action potentials in targeted species (Kuivila *et al.*, 2012; Brander *et al.*, 2016), including fishes (Soderlund, 2012). However, the full modes of action on fish development and behaviour are not fully understood.

Bifenthrin is a lipophilic chemical and thus is likely to absorb to the lipid-rich yolk-sac of larval sturgeon and be assimilated by the developing embryo as it metabolizes these maternal energy stores (Dettlaff *et al.*, 1993). Sturgeon store and digest the yolk intracellularly (in contrast to modern teleosts; Buddington and Doroshov, 1986; Schmalhausen, 1991), yet do not have fully developed organs and enzymes necessary for contaminant detoxification and excretion (gills, liver, kidney). This is a potential mechanism driving the reported increased sensitivity of early life stages to chemical pesticides (Jahanbakhshi *et al.*, 2012), which raises concerns as survival of the young-of-year age class can have a strong influence on population growth rates in sturgeon (Houde, 1989; Gross *et al.*, 2002).

Our goal in the present study was to investigate knowledge gaps specific to the effects of bifenthrin in early life stages of California sturgeon. We tested a range of bifenthrin concentrations that included environmentally relevant concentrations (5–100 ng/l; Amweg *et al.*, 2006, Weston *et al.*, 2019), as well as higher concentrations (500–2000 ng/l) to identify a dose–response curve for these two sturgeon species. We focused on larval sturgeon because we expected larval sturgeon to be more vulnerable to toxicity of bifenthrin, and survival rates of early life stages have outsized consequences for population dynamics. Specifically, we evaluated lethal and sublethal effects of an acute 96-hour exposure of multiple concentrations of bifenthrin by quantifying survival rates and behavioural changes in endogenously feeding green and white sturgeon.

## Materials and Methods

### Study fish

Green sturgeon larvae were obtained from the green sturgeon broodstock held at the Center for Aquatic Biology and Aquaculture (CABA) at the University of California, Davis. The UC Davis broodstock was originated with fertilized eggs from northern Distinct Population Segment adults collected from the Klamath River by the Yurok Tribe. The existing broodstock is maintained through a collaborative and ongoing agreement with the Yurok Tribe. Fish used in this study were obtained from a tank spawning event using one mature female and two mature males, following methods outlined in Van Eenennaam *et al.* (2008). During the spawn, eggs were collected every 4 hours and transferred to upwelling jars where they were incubated at the UC Davis Putah Creek hatchery building with surface water (Lake Berryessa source) at an average of 15°C until hatch. After hatch the larvae were transferred to 450-L circular tanks, also supplied with surface water. Larvae were selected for experiments at 3 days post hatch (dph), and lengths and weights were collected after the exposure period at 7 dph. The mean total length was 18.5 mm (SD = 0.6), and the mean mass was 43.9 mg (SD = 4.8). There was no significant difference in size (total length or mass) between treatment groups at the end of the experiment (Anova: total length, *P* = 0.80; mass, *P* = 0.42).

White sturgeon larvae were obtained from Sterling Caviar (Elverta, CA). Eggs from two mature females, obtained via cesarian section, were combined with milt from five males. Eggs were incubated in upwelling jars at 15°C until hatch. When larvae were 1 dph they were transferred to CABA and held until larvae were selected for experiments at 3 dph. Lengths and weights were collected after the exposure period at 9 dph, as the exposure was extended two additional days to evaluate potential for delayed response. Data presented here are for the initial 96-hour exposure, as no change in mortality or behavioural trends was observed during the additional 48 hours of exposure. The mean total length was 18.6 mm (SD = 0.8), and the mean mass was 44.3 mg (SD = 3.9). There was no significant difference in total length between treatment groups at the end of the experiment (Anova: *P* = 0.11) but there was a significant difference between the control and 1000 ng/l exposure group in mass (Tukey multiple comparisons test: *P* = 0.02). All spawning, rearing and experimental protocols were approved by the University of California Institutional Animal Care and Use Committee protocols #22662 and #21449.

### Bifenthrin exposures

#### Chemical information

The pyrethroid pesticide bifenthrin is one of the most commonly detected pyrethroids in the SFE, frequently at aqueous concentrations at or above known toxicity levels (Amweg *et al.*, 2006; Sanders *et al.*, 2018; Weston *et al.*, 2019). Aqueous bifenthrin exposures were mixed using bifenthrin powder (ChemService, West Chester, PA, USA. CAS: 82657–04-3, product #: N-11203-100MG) dissolved in methanol as a solvent vehicle to form a concentrated stock of bifenthrin (10 mg/l). Larvae were exposed to a vehicle control and five nominal concentrations of bifenthrin: 10, 100, 500, 1000 or 2000 ng/l. Each water mixture, including solvent controls, contained the same concentration of the methanol vehicle (0.2 ml/l). The range of bifenthrin concentrations was selected to include environmentally relevant concentrations measured in surface waters (5–100 ng/l; Amweg *et al.*, 2006; Weston *et al.*, 2019), as well as higher concentrations (500–2000 ng/l) to describe a dose–response curve for comparative purposes across these two sturgeon species.

The aqueous concentrations of exposure waters were verified through chemical analysis (Table 1). Water samples, method blanks and lab spikes were spiked with an isotope-labelled surrogate, Chlorpyrifos D10, as a quality control prior to extraction. Samples were passed through a solid phase extraction cartridge (Oasis HLB, Waters) and eluted with 10 ml ethyl acetate. The empty sample glass containers were rinsed with hexane/acetone (3/1, v/v) and combined with the eluent. The combined extracts were concentrated to 0.2 ml and 4,4′-dibromooctafluorobiphenyl (DBOFB) was spiked as the internal standard prior to analysis using gas chromatography with negative chemical ionization (GC-NCI) analysis following previously published methods (Moschet *et al.*, 2017; Budd *et al.*, 2023). Across multiple analytical sample batches, the highest limit of detection for bifenthrin was 0.2 ng/l.

**Table 1:** Aqueous exposure concentrations of bifenthrin, as determined through chemical analysis prior to exposure

	Nominal concentration (ng/l)					
	0	5	100	500	1000	2000
Green sturgeon exposures	ND	2.5	69.6	472.5	724.9	2117.6
White sturgeon exposures	ND	0.6	69.6	301.8	632.7	1985.9

Concentrations measured in ng/l. Highest limit of detection (LOD) = 0.2 ng/l; ND = not detected.

#### Exposure procedures

To initiate each experiment, exposure waters were prepared using untreated surface water and the concentrated stock of bifenthrin. Exposure water of each nominal concentration was added to four replicated large glass containers (1000 ml volume, 19 cm diameter) and eight replicated small glass containers (100 ml volume, 7 cm diameter), which were then placed into a flow-through water bath to maintain the exposure water at testing temperature (15°C). Each dish was equipped with a glass aeration tube to maintain suitable levels of dissolved oxygen. After containers were prepared, 3 dph larvae were added. During green sturgeon experiments, large containers contained 20 larvae and were used to estimate mortality rates, while small containers contained one larva and were used to evaluate behavioural changes. During white sturgeon experiments, large containers contained 50 larvae, and small containers contained one larva. On each day of the 96-hour exposure period, 50% of the water in each container was removed and replaced to maintain water quality and exposure concentrations. Replacement water was mixed to the target exposure concentrations following the same methods used for experiment initiation. Any mortality was recorded in the large exposure containers when water was changed.

#### Behavioural evaluation

After 96 hours of exposure, following the standard length for acute toxicity exposures, the exposure containers holding single individuals were moved to a video recording table to evaluate sublethal behavioural effects of aqueous exposure to bifenthrin. Light was supplied from underneath the dishes (US Art Supply, Lightmaster) and cameras (ActiveOn, CX; Narrow POV, 30 fps) were mounted above to record behaviour. These videos were post-processed using Ethovision Software (Noldus, Ethovision XT15) to quantify behaviour of individual larvae. Ethovision provided the position of the fish in each frame during the 12-minute video recording, analysed at 30 frames per second (fps). Initiation of data collection occurred after three minutes of video recording because a visual assessment of trends in the data showed that activity increased in this first interval of time then reached a plateau for the remainder of the trial. After acquisition of the fish positions, the tracks were filtered by removing inter-position movements less than 0.2 mm and greater than 7.5 mm. Short movements were likely to result from tiny adjustments in the automatic tracking, while long movements were considered biologically unlikely (>12 body lengths per second) and this threshold effectively removed most large-scale errors. After filtering, tracks were manually edited to remove any remaining erroneous detections. A central zone was also defined within the analysis software as a circular area centered within arena, having a diameter of 4 cm and covering 33% of total arena area. Each recorded position was classified as inside or outside this central zone. After filtering and editing, movement data were summarized to five positions per second by calculating the sum of distance moved and sum of relative turning angle across all positions occurring within each 0.2-second interval. Each summarized position was classified as occurring inside the central zone if any recorded positions were within the zone.

Using the acquired and filtered tracks, a suite of movement parameters was estimated for each fish after 96 hours of exposure: the total distance traversed (TDT), the mean meander (absolute turning angle between subsequent positions, standardized by distance traversed; MM), and percentage of time spent within the central zone of the arena (PTCZ). TDT was evaluated as an indicator of activity level, with implications for movement-related energy expenditure and potential for foraging. Previous work with contaminant exposure in fishes has shown that neurotoxic pesticides can reduce distance travelled by exposed individuals (Floyd *et al.*, 2008; Mundy *et al.*, 2021; Mauduit *et al.*, 2023). MM is often used as an indicator of movement coordination, reflecting erratic swimming that can result from neurotoxic effects of pesticide exposure (Faria *et al.*, 2018, Biswas *et al.*, 2018, Mundy *et al.*, 2020). PTCZ was also selected for evaluation as low PTCZ reflects high thigmotaxis, the behavioural attraction to the edges of a holding chamber. In zebrafish models thigmotactic behaviour is related to anxiety and it can decrease when contaminants or other interventions reduce anxiety-related responses (Richendrfer *et al.*, 2012). Alternatively, for fish such as sturgeon, which typically show high levels of thigmotaxis (Pavlov and Kasumyan, 2000), a reduction in thigmotactic behaviour may result from a loss of muscle coordination due to neurotoxic effects of pesticide exposure (Jin *et al.*, 2009; DeMicco *et al.*, 2010). We expected that changes in overall activity would be reflected in TDT, while changes in swimming coordination or capacity would be reflected in MM and PTCZ.

#### Statistical analysis

A Pearson’s pairwise partial correlation test was conducted on all two-way comparisons of movement parameters (TDT, MM, PTCZ) collected from individually exposed larvae, controlling for species and bifenthrin exposure concentration (Kim, 2015). To assess sublethal effects of bifenthrin exposure, independent models were then built for each movement parameter. Sample size varied across treatments (Table 2), as some replicates were excluded from analysis due to late-developing deformities (5 of 48 trials) or filtering to remove trials with low tracking success (<66% positions, 6 of 48 trials). For the two positive continuous response variables (TDT and MM), measured bifenthrin concentration, fish mass (centered) and species, were used as predictor variables within Gaussian models. Species was allowed to interact with bifenthrin concentration, as we anticipated species-specific responses to exposure, and species also interacted with mass as white sturgeon were two days older than green sturgeon when measured. Diagnostic plots were reviewed for each selected model to ensure the model assumptions were met. When they were not, logarithmic transformations were explored for bifenthrin concentration and for the response variables to identify the model form that best met linear model assumptions. The explanatory value of the interaction terms were evaluated using AIC and excluded from the selected model if they did not provide additional information (i.e. the interaction reduced AIC value by less than 2 points).

**Table 2:** Number of trials used in statistical analyses for each movement parameter

Species	Movement parameter	Nominal concentration (ng/l)					
		0	5	100	500	1000	2000
Green sturgeon	TDT	7	7	8	6	7	8
	MM	7	7	8	6	7	8
	PTCZ	6	6	7	4	7	7
White sturgeon	TDT	8	8	8	8	8	8
	MM	8	8	8	8	8	8
	PTCZ	8	8	8	8	8	8

Each trial included a single green sturgeon larvae after 96-hour exposure period.

To predict the PTCZ, a beta regression model was selected as this model form is better suited for continuous data bounded at 0 and 1 (i.e. percentage data). The model was fit with a logit link function of the mean response and a log link function of the precision term. Six trials with green sturgeon were dropped from the analysis because they were missing more than one third of potential detections due to problems with video quality and automated tracking success (Table 2). The predictor variables considered within the model were the same as above, and the precision term was allowed to vary between species. The explanatory value of the species by bifenthrin interaction term was evaluated using AIC and excluded if it did not provide additional information. These three models evaluated behavioural differences between species under control conditions, as well as allowing us to compare predictions and 95% confidence intervals (delta method) within species at each of the bifenthrin concentrations tested.

## Results

Overall, the survival was high (~95%) after the 96-hour exposure period for both species at all bifenthrin concentrations and control groups, validating the exposure test and confirming the concentrations tested were within the sublethal range for aqueous exposures (Supplemental Information, Fig. S1). The partial pairwise correlations of movement parameters showed statistical significance (all *P* < 0.01), but all correlations were less than 0.35 (Table S1).

### Total distance traversed

**Figure 1:**
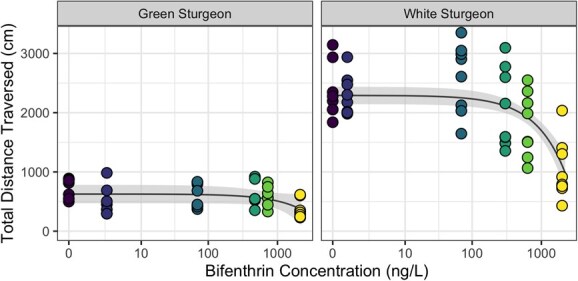
Relationship between total distance traversed and measured aqueous concentration of bifenthrin for larvae of green and white sturgeon. Movement paths were quantified for larvae at 7 days post hatch, after 96-hour exposure. Each point indicates total distance travelled by an individual larvae, as calculated from tracks obtained via automated detection software (*n* = 6–8 per treatment), while the line shows the predicted relationship from the general linear model for a fish of mean mass. The ribbon indicates the 95% confidence interval of the predicted relationship

The linear model selected to predict total distance traversed (cm; TDT) included predictive terms for species, bifenthrin concentration and mass, as well as both interaction terms for species. Reported predictions are for fish of mean mass (43.5 mg). Under control conditions, white sturgeon were predicted to travel significantly farther than green sturgeon (difference of 1666 cm, SE = 107, *P* < 0.001; Fig. 1, Table S2). White sturgeon also showed a significant reduction in TDT under increasing concentrations of bifenthrin (*P* < 0.0001), with an estimated decrease of 6.0 cm (SE = 0.86) in distance traversed for every 10 ng/l increase in bifenthrin concentration. This results in a predicted TDT of only 1102 cm (95% CI, 828–1376) when white sturgeon larvae were exposed to 2000 ng/l bifenthrin for 96 hours. This is in contrast to the predicted TDT of 2292 cm (95% CI, 2146–2439) for white sturgeon larvae under control conditions. However, the effect of bifenthrin was not statistically significant for green sturgeon (*P* = 0.127), for which the model predicted a decrease in TDT of only 1.2 cm (SE = 0.77) for every 10 ng/l increase in bifenthrin concentration. This results in a predicted TDT of 390 cm (95% CI, 145–634) when green sturgeon larvae were exposed to 2000 ng/l bifenthrin for 96 hours, as compared to a predicted TDT of 627 cm (95% CI, 471–782) for green sturgeon under control conditions.

**Figure 2:**
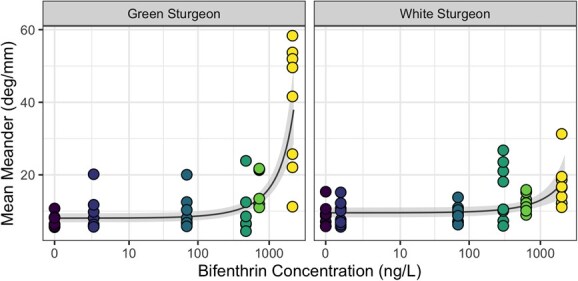
Relationship between mean meander of an observed movement path and measured aqueous concentration of bifenthrin for larvae of green and white sturgeon. Movement paths were quantified for larvae at 7 days post hatch, after 96-hour exposure. Points indicate results from automated detection software for individual larva within replicated trials (*n* = 6–8), while the line shows the predicted relationship from the general linear model. The ribbon indicates the 95% confidence interval of the predicted relationship

### Mean meander

The linear model selected to predict the mean meander (deg/mm; MM) included a logarithmic transformation of the response variable and the untransformed predictive terms for species, bifenthrin concentration and their interaction. Mass was not an important predictor for this movement parameter and was excluded from the model. Under conditions without bifenthrin present there was no significant difference in MM between the two sturgeon species (effect size: 1.18 deg/mm, SE = 1.11, *P* = 0.119; Fig. 2, Table S3). The meandering behaviour of both species was significantly affected by increasing bifenthrin (GS: *P* < 0.0001, WS: *P* = 0.0004), yet the species differed in the strength of that response. Green sturgeon increased their MM significantly more than white sturgeon as bifenthrin levels increased (*P* = 0.001). Predicted MM for the track of a green sturgeon larvae exposed to 2000 ng/l after 96 hours was 33.1 deg/mm (95% CI, 25.5–42.5), as compared to a predicted MM of 8.1 deg/mm (95% CI, 6.9–9.4) under control conditions. White sturgeon were predicted to meander only 17.8 deg/mm (95% CI, 13.5–23.3), as compared to a predicted MM of 9.6 deg/mm (95% CI, 8.3–11.0) under control conditions. Mean meander was calculated from absolute turning angles at each step, and thus both small-scale body undulations, as well as large-scale movements are reflected in this metric.

### Percent time spent in the central zone

**Figure 3:**
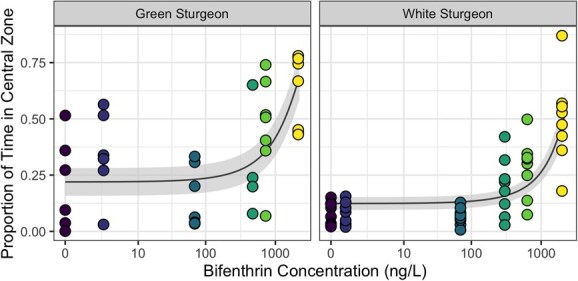
Relationship between the percentage of time spent in the central zone of the testing arena and measured aqueous concentration of bifenthrin for larvae of green and white sturgeon. Locations within the arena were quantified for larvae at 7 days post hatch, after 96-hour exposure. Points indicate results from automated detection software for individual larva within replicated trials (*n* = 4–8), while the line shows the predicted relationship from the general linear model. The ribbon indicates the 95% confidence interval of the predicted relationship

The beta regression model selected to predict the percentage of time a larvae spent within the central zone of the arena (PTCZ) included species and bifenthrin concentration, but their interaction was excluded during model selection. Mass and its interaction with species were also included, based on AIC evaluation. Reported predictions are for fish of mean mass (43.5 mg). Under conditions without bifenthrin present, green sturgeon had significantly greater PTCZ than white sturgeon (effect size: 9.9%, SE = 2.9, *P* < 0.001; Fig. 3, Table S4). Both species responded to increasing concentrations of bifenthrin (*P* < 0.0001), with no statistically significant difference between species in the strength of that response. After exposure to 2000 ng/l of bifenthrin for 96 hours, the model predicted a PTCZ for green sturgeon larvae of 60.5% (95% CI, 50.7–70.4), as compared to a predicted PTCZ of 19.8% (95% CI, 14.3–25.3) under control conditions. For white sturgeon, the model predicted a PTCZ of 47.0% (95% CI, 38.4–55.6) after exposure to 2000 ng/l bifenthrin for 96 hours, as compared to a predicted PTCZ of 12.5% (95% CI, 9.6–15.3) under control conditions.

## Discussion

In the present study, we observed low mortality for both green and white sturgeon larvae after acute aqueous exposure to bifenthrin at and above environmentally relevant concentrations (5–2000 ng/l; Amweg *et al.*, 2006, Weston *et al.*, 2019). We also observed significant sublethal effects on locomotion following bifenthrin exposure in both species. For all three locomotion parameters considered, total distance traversed (TDT), mean meander (MM) and percent of time spent in the central zone of the arena (PCTZ), larvae exposed to the 2000 ng/l concentration of bifenthrin showed distinctly different behaviours than the control groups, although these trends were not always statistically significant. The strength of the effect and the threshold above which our statistical models predicted a significant change varied by species and movement metric, yet it is clear that locomotion of larval sturgeon is affected by bifenthrin at high concentrations. One potential mechanism resulting in these trends could be inhibition of muscle coordination of the larvae (Kuivila *et al.*, 2012; Soderlund, 2012), reducing overall activity, increasing erratic movements and preventing larvae from selecting preferred regions within the testing arena. Subjective observations of erratic movement patterns and muscle spasms in larvae exposed at 2000 ng/l further support this hypothesis. However other mechanisms could also lead to altered behaviour, such as energetic reallocation or changes in genome expression (Ingham *et al.*, 2021; Lucero and Muñoz-Quezada, 2021). Regardless of the mechanism, the observed sublethal behavioural effects can indirectly reduce recruitment of sturgeon larvae to juvenile life stages due to increased predation risk or decreased foraging efficiency (Floyd *et al.*, 2008; Frank *et al.*, 2019) and thus are relevant for conservation management of species of concern such as sturgeons.

Total distance traversed was used as an estimate of activity level and is a common method for quantifying locomotion and energy use in behavioural and toxicological studies. Previous work has shown that bifenthrin can alter activity levels of fishes after aqueous exposure. Both larval fathead minnows (*Pimephales promelas*) and larval longfin smelt (*Spirinchus thaleichthys*) reduced activity levels after exposure to the pyrethroids esfenvalerate and bifenthrin, respectively (Floyd *et al.*, 2008; Mauduit *et al.*, 2023), while larval Delta smelt (*Hypomesus transpacificus*) have shown hyperactivity after exposure to low or moderate concentrations of bifenthrin (2–100 ng/l; Mundy *et al.*, 2020). For larval white sturgeon in the current study, there was a weak, non-significant trend for hyperactivity at intermediate concentrations (100 ng/l) and strong and significant evidence that bifenthrin reduced activity levels at high concentrations (≥500 ng/l). For green sturgeon we saw similar trends but no significant differences in activity levels across treatments. This lack of statistical significance may be because changes in activity were more difficult to detect, given the lower overall movement of green sturgeon larvae at this life stage (Kynard *et al.*, 2005; Kynard and Parker, 2005). Altered activity levels can have implications for foraging success and predation risk (Floyd *et al.*, 2008, Frank *et al.*, 2019) and may indirectly impact population persistence if effects are widespread.

Path meander is often used to measure erratic swimming (Orger and de Polavieja, 2017; Razali *et al.*, 2022) and has been shown to increase when fish are exposed to contaminants that induce neurotoxicity (Biswas *et al.*, 2018; Faria *et al.*, 2018). Our qualitative observations suggested that under high bifenthrin concentrations (1000 and 2000 ng/l) sturgeon larvae alternated between periods of resting and periods marked with erratic, jerking movements suggestive of loss of muscle control. Often these locomotor events did not achieve directional movement, especially at the highest bifenthrin concentration. This locomotion pattern contrasted with larvae observed under control conditions, where sturgeon movement was characterized by periods of rest interspersed with active swimming in a broad circle around the edge of the arena with a slight ‘wobble’ due to the continued presence of the yolk sac at 96 hours of exposure (7 dph). MM measurements reflected these qualitative observations, showing a positive relationship between MM and bifenthrin concentration. The model predictions showed an effect of bifenthrin for both green and white sturgeon, where larvae exposed to ≥500 ng/l or ≥1000 ng/l, respectively, had greater meander than larvae in lower concentration treatments, with no overlap in prediction confidence intervals. These results suggest that at high concentrations bifenthrin may have neurotoxic effects and impair muscle control in larval sturgeon, although reallocation of energy could also alter behaviours. Future studies of AChE levels or other molecular markers would help identify the mechanisms leading to these sublethal effects.

Green and white sturgeon are benthically oriented fish and are highly thigmotactic, preferring to rest along the edges of a testing arena (Pavlov and Kasumyan, 2000). This behaviour was observed in sturgeon larvae under control conditions, resulting in fewer than half of the recorded positions occurring within the centre zone of the arena. However, larvae exposed to higher concentrations of bifenthrin showed increasing use of the central zone. Qualitative observations of behaviour in the high concentration treatments suggested the increase in use of the central zone may have been related to erratic movements and stationary spasms. Spasms were defined as contractions of the larvae into a C-curve, occurring on alternating sides with no subsequent directional movement. Model predictions confirmed that for both species, larvae exposed to ≥1000 ng/l were more likely to be in the centre of the arena than larvae exposed to ≤100 ng/l, with no overlap in confidence intervals of the predictions. While the mechanism was not directly tested, this increasing percentage of time spent within the central zone (PTCZ) may have resulted from a loss of muscle coordination and corresponding reduction in volitional movement to preferred locations at the edge of the arena. Pyrethroid pesticides disrupt the function of sodium channels in fish, delaying the inactivation of the voltage gated channels and resulting in neuronal excitability (Ullah *et al.*, 2019). Zebrafish exposed to sublethal concentrations of bifenthrin during early development showed an increase in spontaneous movements and loss of muscle coordination (Jin *et al.*, 2009). Yet when zebrafish embryos were exposed to both a pyrethroid pesticide and a sodium channel agonist (MS-222), the incidence of muscle spasms was decreased and the larvae could swim normally in response to a stimuli, suggesting the spasms were neuronal in origin (DeMicco *et al.*, 2010). Thus, it is plausible that in sturgeon larvae the increased frequency of spasms and subsequent increase in PTCZ was due to neuronal disruption and loss of motor control after the acute exposure period.

We saw the strongest and most consistent sublethal effects of bifenthrin when larvae were exposed to 2000 ng/l of bifenthrin. Runoff from residential areas contained bifenthrin at concentrations as high as 6120 ng/l, as measured at storm water drainage outflows (Beggel *et al.*, 2011). It is unlikely this concentration would commonly be encountered in areas where these larval sturgeons develop as they prefer to spawn in large river systems where high concentration run-off is diluted to lower concentrations. However, bifenthrin has been observed at higher aqueous concentrations in the San Joaquin than the Sacramento River (Weston and Lydy, 2010) and may pose a greater threat to the intermittent and smaller spawning populations of sturgeon in the San Joaquin River.

While the present study tested only aqueous exposure of endogenously feeding fish, it is important to note that bifenthrin and other pyrethroid pesticides are lipophilic and bind to sediment and suspended solids in natural waterways, as well as on aquatic organisms. In a mesocosm study, bifenthrin accumulated in sediments under a program of weekly dosages (Pennington *et al.*, 2014), and sediments have been shown to be toxic after pyrethroid accumulation (Ding *et al.*, 2010). Thus, benthically oriented larvae and juveniles may encounter greater exposure from sediment contact within boundary habitats than from aqueous exposure alone. Additionally, the present study did not consider effects on exogenously feeding life stages, nor potential for bioconcentration via consumption of contaminated prey. *Hylella azteca* in the Central Valley of California have developed resistance to pyrethroid pesticides and were shown to bioaccumulate bifenthrin, up to 1000-fold higher levels than non-resistant clades (Johanif *et al.*, 2021), suggesting important avenues of exposure for higher trophic organisms. Pyrethroid pesticides are lipophilic and accumulate in the food web at magnitudes higher than expected through direct aqueous exposure (Huff Hartz *et al.*, 2021; Magnuson *et al.*, 2022; Xie *et al.*, 2022). Bifenthrin has been shown to bioaccumulate in fish with a reported body burden approximating 4500 ng/kg (Miranda *et al.*, 2022) to 8890 ng/kg (Xie *et al.*, 2022). Bioaccumulation can also lead to maternal transfer to eggs and offspring (Metts *et al.*, 2013), and pyrethroid exposure during development has been correlated with increased rates of neurodevelopmental disorders (Furlong *et al.*, 2017). Thus, it would be valuable for future work to explore sturgeon responses to sediment-bound pesticides and contaminated prey, verify pesticide body residues, assess rates of maternal transfer and ascertain effects from resulting body burden. Greater body burdens can result in increased mortality rates as well as increased energetic costs of detoxification with indirect impacts that may be important for species of conservation concern, such as sturgeons.

In summary, this study shows that while acute (96 hours) lethal effects of bifenthrin are limited at environmentally relevant aqueous concentrations, sturgeon experience sublethal effects on locomotion under direct exposure to higher bifenthrin concentrations (500–2000 ng/l). Impaired movement can have indirect effects on cohort success through increased predation risk or decreased foraging efficiency (Floyd *et al.*, 2008). Increased mortality during this early life stage can impair recruitment, with decadal consequences for these long-lived species. More broadly, these results expand our understanding of threats to the persistence and recruitment of green and white sturgeon. To ensure success of future recovery planning and management actions, continued studies of singular and interacting stressors will help us to gain a practical understanding of modes of toxicity and the ecological effects of ubiquitous contaminants.

## Supplementary Material

Web_Material_coaf055

## Data Availability

All data used in the present manuscript are freely available at the online repository “Environmental Data Initiative” within the data package titled “Effects of Multiple Stressors on Ecological Performance of Early Life Stage Sturgeon” (edi.1621.1). https://doi.org/10.6073/pasta/b0646fb9f2d546c7032a9911d7493ef1
