# Supplementary material for: Aqueous exposure to a pyrethroid pesticide results in behavioural effects in early life stage sturgeon
Source: Conserv Physiol. 2025 Jul 30;13(1):coaf055. doi: 10.1093/conphys/coaf055 (PMC12310246; doi:10.1093/conphys/coaf055)
Supplement: Web_Material_coaf055 [file web_material_coaf055.pdf]

Aqueous Exposure to a Pyrethroid Pesticide Results in Behavioral Effects in Early Life Stage Sturgeon

Anna E. Steel[1], Sarah E. Baird[1], Dennis E. Cocherell[1], Thomas M. Young[2], Richard E. Connon[3], Nann A. Fangue[1]

[1]Department of Wildlife, Fish, and Conservation Biology, University of California, Davis
[2]Department of Civil and Environmental Engineering, University of California, Davis
[3] School of Veterinary Medicine, Department of Anatomy, Physiology and Cell Biology, University of California Davis

Supplemental Material

Acute mortality rates

Binomial models with a logit link function were built to describe mortality rates, using measured concentrations of bifenthrin, species, and their interaction, as predictor variables. Diagnostic plots were reviewed for each selected model to ensure the model assumptions were met. The explanatory value of the interaction term was evaluated by assessing the change in AIC for models with and without the interaction. Diagnostic plots were reviewed for each selected model to ensure the model assumptions were met.

Overall, there were low (<6%), non-significant mortality rates after the 96 h exposure period for both species at all controls and bifenthrin concentrations (Figure S1). The general linear model fit to these data indicated that there was a significant effect of bifenthrin concentration on mortality for both species, and a significant difference between species. However, the size of the effect was predicted to be very small; for green sturgeon under control conditions the model predicts 1.1% mortality and for those exposed at 2000ng/L it predicts 4.2% mortality. For white sturgeon these predictions were even smaller, with mortality under control conditions predicted to be 0.3% and mortality at 2000 ng/L to be 1.0%.

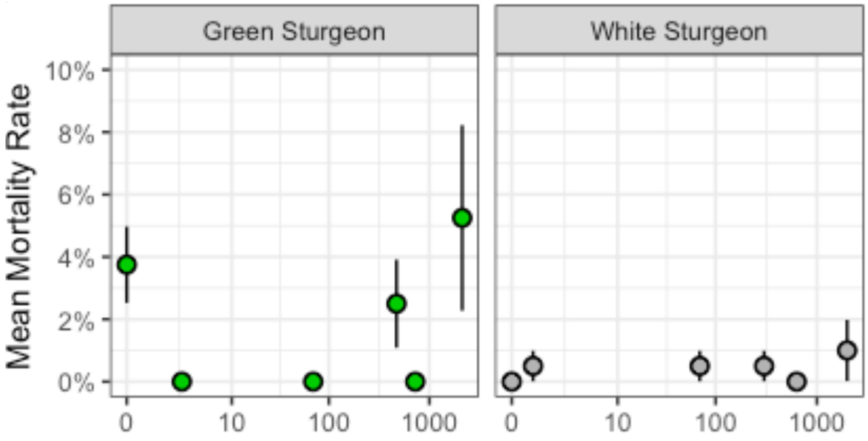

Figure S1. Acute mortality rates observed for larval green sturgeon (GS) and white sturgeon (WS) after 96 h of exposure to experimental concentrations of the pesticide bifenthrin. Points indicate mean estimates across four replicated exposure groups (N_{GS} = 20, N_{WS} = 50). Lines indicate the 95% confidence interval around the mean.

Partial Pairwise Correlations of Movement Parameters

Table S1. Pearson's partial pairwise correlation tests were conducted for all pairwise combinations of the three movement parameters used in the subsequent analysis (Total Distance Traveled, log transformation of Mean Meander, and Percent Time spent in the Central Zone). The correlation tests controlled for covariates of species and bifenthrin exposure concentrations. For all comparisons, n=91.

Pairwise behavioral metrics	Partial pairwise correlation estimate	p-value	t-statistic
Total Distance Traveled by log(Mean Meander)	-0.280	0.008	-2.718
Total Distance Traveled by Percent Time in Central Zone	-0.263	0.013	-2.538
Percent Time in Central Zone by log(Mean Meander)	0.273	0.010	2.643

Model Predictions for Sublethal Effects

Table S2. Model predictions and 95% confidence interval of total distance traversed (TDT) by a larval sturgeon after 96 h exposure to bifenthrin. Distance was calculated for replicated trials (n=6-8) at each concentration using computer automated detection software.

	Bifenthrin Concentration	Estimated TDT (cm)	95% Confidence Interval	n trials
Green Sturgeon Exposures	0	636	[484, 787]	7
	5	635	[484, 786]	7
	100	624	[481, 766]	8
	500	575	[453, 696]	6
	1000	514	[378, 650]	7
	2000	392	[142, 642]	8
White Sturgeon Exposures	0	2374	[2234, 2514]	8
	5	2371	[2231, 2510]	8
	100	2305	[2174, 2436]	8
	500	2028	[1913, 2142]	8
	1000	1681	[1541, 1821]	8
	2000	988	[718, 1258]	8

Table S2. Model predictions and 95% CI of the mean meander (degrees turned per mm traversed) displayed by a larval sturgeon after 96 h exposure to bifenthrin. Meander was calculated for replicated trials (n=6-8) at each concentration using computer automated detection software, and the mean meander was calculated for each replicate. Tracks were not smoothed prior to estimation of meander, and therefore the metric incorporates body undulations as well as large-scale turning behavior.

	Bifenthrin Concentration	Estimated Mean Meander (deg/mm)811.	95% Confidence Interval	n trials
Green Sturgeon	0	8.08	[6.89, 9.39]	7
	5	8.08	[6.96, 9.39]	7
	100	8.67	[7.46, 9.97]	8
	500	11.47	[10.18, 12.94]	6
	1000	16.28	[14.15, 18.73]	7
	2000	33.12	[25.53, 42.52]	8
White Sturgeon	0	9.58	[8.25, 11.02]	8
	5	9.58	[8.33, 11.02]	8
	100	9.87	[8.58, 11.25]	8
	500	11.13	[9.97, 12.55]	8
	1000	13.07	[11.25, 15.03]	8
	2000	17.81	[13.46, 23.34]	8

Table S3. Model predictions and 95% CI of the percentage of time spent within the central zone of the testing arena after 96 h exposure to bifenthrin. Locations within the arena was calculated for replicated trials (n=4-8) at each concentration using computer automated detection software.

	Nominal Bifenthrin Concentration	Estimated % time in Central Zone	95% Confidence Interval	n trials
Green Sturgeon	0	19.8	[14.3, 25.3]	6
	5	19.9	[14.4, 25.3]	6
	100	21.3	[15.7, 26.9]	7
	500	28.0	[21.8, 34.3]	4
	1000	38.1	[30.8, 45.4]	7
	2000	60.5	[50.7, 70.4]	7
White Sturgeon	0	12.5	[9.6, 15.3]	8
	5	12.5	[9.7, 15.4]	8
	100	13.5	[10.6, 16.4]	8
	500	18.4	[15.2, 21.6]	8
	1000	26.2	[22, 30.4]	8
	2000	47.0	[38.4, 55.6]	8
